# Virtual Reality Assisted Non-Pharmacological Treatments in Chronic Pain Management: A Systematic Review and Quantitative Meta-Analysis

**DOI:** 10.3390/ijerph19074071

**Published:** 2022-03-29

**Authors:** Simone Grassini

**Affiliations:** 1 Department of Social Studies, University of Stavanger, 4021 Stavanger, Norway; simone.grassini@uis.no; 2 Department of Psychology, NTNU–Norwegian University of Science and Technology, 7491 Trondheim, Norway

**Keywords:** virtual reality, chronic pain, pain management, neck pain, back pain

## Abstract

Virtual reality (VR) is a developing technology that has recently attracted the attention of healthcare practitioners. Recently, VR systems have been used to treat pain symptoms. The present study aims to evaluate the VR effectiveness on chronic pain management. A systematic literature search was performed following the Preferred Reporting Items for Systematic Reviews and Meta-Analyses (PRISMA) guidelines. Keywords were used to discover the potentially eligible studies. The primary focus of the present investigation was to evaluate the possible effect of VR-assisted treatments on chronic pain, especially in the commonly occurring low back and neck pain. Nine studies reporting randomized controlled trials were included in the present study. VR-mediated interventions demonstrated significant improvement for pain symptoms in patients experiencing chronic pain. In addition, VR-mediated therapy decreased pain intensity and disability in the case of chronic neck pain compared to control conditions. However, the VR interventions showed a statistically non-significant improvement in chronic low back pain when experimental groups were compared with controls. VR therapy positive effect on chronic pain did not differ from the one reported for other types of interventions for pain management, as physical exercise and laser therapy. Taken together, these findings showed that currently available lines of evidence on the effect of VR-mediated therapy in chronic pain management, despite pointing towards possible therapeutical benefits of the VR-based intervention, are overall inconclusive and that more research on VR-assisted therapy for chronic pain is needed.

## 1. Introduction

The scientific literature has defined pain as an “unpleasant sensory and emotional experience associated with actual or potential tissue damage or described in terms of such damage” [1] (also see recent development of the definition as discussed in [2]). Pain has a negative impact on individual social and psychological functioning that reduces the quality of life [3,4].

Pain can be managed utilizing both non-pharmacological and pharmacological intervention. One of the most reported types of pain is the chronic low back pain (CLBP), and people experiencing CLBP usually seeks medical attention for pain management prescription [5,6]. Within the pharmacological approach, non-steroid anti-inflammatory drugs (NSAIDs) and opioids are usually prescribed [7,8]. Despite its therapeutic effectiveness, this approach has significant drawbacks. Opioids cause tolerance, dependency, and hyperalgesia [9]. In addition, some analgesics require invasive therapy, such as intrathecal administration of drugs that have their own set of clinical risks and complications [10]. A non-pharmacological approach, such as physiotherapy and physical exercise, have demonstrated to provide pain relief and functional improvement [11]. Some other non-pharmaceutical interventions, for example, the application of hot or cold packing in the area where pain is experienced, as well as electric nerve stimulation, have also been questioned for their efficacy in pain management [12,13]. However, the most significant barriers in most non-pharmacological therapy, such as physical exercise, is the lack motivation and the ability of a patient to adhere to the therapeutic prescriptions [14]. The use of modern technological solutions for therapeutic purposes can overcome these barriers by addition motivational components and involvement in therapy for some users. Virtual reality (VR) is a solution that is gaining attention as non-pharmaceutical therapy for pain management [15]. VR entertainment and interactive aspects and feedback can boost adherence to activities [16], and immersive VR systems have been shown to provide pain distraction methods that provide highly engaging external stimuli with the aim to reduce pain by directing user attention to a virtualized environment [17,18]. The use of such types of VR for training and exercise is often referred to in the literature as exergaming and has been shown in the scientific literature to promote physical activity levels (for a review see the work of Sween et al. [19]). The most frequent application of VR systems in clinical practice has been, among others, in pediatric movement disorder rehabilitation [20], elderly neurodegenerative movement disorder rehabilitation [21], post-stroke rehabilitation [22], and for psychiatric disorders [23].

VR systems generally enable user interaction to virtual environments to feel real, via a phenomenon often referred as “sense of presence”, often investigated in psychological research [24,25,26]. Users experience virtual environments using custom made devices, hardware, and software. VR can be immersive, semi-immersive or non-immersive, based on desired stimulated physiological senses, artificial stimuli reliability, degree of interaction with VR environment, and isolation of the users from the external environment [27,28].

Users generally experience VR, in the context of modern immersive visual technology systems, using a head-mounted display (HMDs) and hand controllers, and sensors detecting head and body movements. VR systems are readily available, easily portable, and—especially in its latest consumer-oriented iterations—affordable to the public. Thus, physicians have considered VR systems as a novel therapeutic option, able to provide analgesic effects [29].

Even though several studies have assessed VR use in acute and chronic pain management, none of the studies clearly explained the exact mechanism of pain relief for VR. However, a few studies hypothesized distraction as a possible mechanism for VR effects on pain alleviation [30]. Usually, VR causes psychological distraction from pain stimuli by viewing a captivating video or playing a game. Patients experience less pain and increased pain tolerance and mood due to the attention shift [31]. The possibility to effectively use VR for pain management in chronic pain has attracted the attention of the scientific literature in the last few years (see, e.g., [32,33,34]). Recently published systematic review articles have also focused on understanding the efficacy of VR-based interventions in the context of pain management (for example, see [35]). However, recent systematic efforts to evaluate the efficacy of VR for chronic pain management specifically for the most reported chronic pain forms are still missing. A notable exception is the study by Mallari et al. [36], however, that study, quantitatively analyzed only a small number of nowadays outdated scientific studies and did not focus specifically on chronic pain). The present literature review and meta-analysis aims to fill the literature gap and to provide a quantitative evaluation of published studies that have employed the most updated types of VR. This effort will help towards an evidence-based assessment of the efficacy of the use of VR for chronic pain management from the perspective of establishing health recommendations and best practices.

## 2. Method

### 2.1. Design

The present study focuses on estimating the overall effect of the use of VR on chronic pain management. The study follows the recommendations of “Preferred Reporting Items for Systematic Review and Meta-analysis (PRISMA)” [37].

### 2.2. Search Strategy and Database

Several databases of scientific literature were explored, with a focus on performing research on databases specializing in clinical and health research. The time-window of publications that was considered was between January 2016 and December 2021, and research was performed using the following medically oriented databases of scientific literature: PubMed, APA Pyschoinfo, CINAHL, clinicaltrail.gov, Cochrane Library, and Embase. The time-window for the literature search was selected to include studies that made use of modern VR systems and excluded old technological solutions that may not be representative of current technology.

An electronic search was performed attempting to discover potential articles using the following keywords and MESH terms: virtual reality, virtual reality therapy, immersion, virtual environment, VR, pain, wound, burn, and surgery. Boolean operators and relevant keywords were used. In addition, search keywords were matched based on a different database.

### 2.3. Study Criteria

Only articles that met the following study criteria were included: (1) randomized controlled trials (RCTs); (2) chronic pain patients or pain lasting longer than 3 months; (3) RCT with VR intervention; (4) RCT exploring usual care or traditional therapy in one of the study arms; (5) RCT assessing any pain outcomes; and (6) patients experiencing chronic pain neck or back pain. Research of the articles was restricted to human patients and the English language. Chronic back and neck pain were selected as a study focus as they were commonly reported conditions, have been relatively well studied in the scientific literature—especially in the context of pain management (see, e.g., [38,39]), and have high clinical and economical [40].

Articles were excluded if: (1) they were review articles (no primary data); (2) no full text was available; (3) they were editorial letter/commentaries; (4) they were non-research letters; (5) they were case reports or case series; (6) included patients with acute pain or pain induced from cold-pressors; (7) were conference abstracts; (8) were articles without quantitative measurement of pain and analgesic effects; or (9) were studies including an insufficient number of participants (this was established to be five patients).

### 2.4. Study Selection, Data Extraction and Quality Assessment

Each identified article was evaluated individually to eliminate duplicates that did not meet the study criteria. A full-text review was completed if the abstract of relevant articles could not demonstrate specific results. Full texts were evaluated based on study exclusion and inclusion criteria. Data extraction was performed after full-text analysis was added to a data-collection form using Microsoft word. The study author extracted article details, such as study design, the sample size in intervention and control group, mean age, pain type, VR environments, pain location treatment condition, pain assessment method, and a summary of each article. The Newcastle–Ottawa Scale (NOS) was used to measure the bias of the study [41]. Only articles with NOS greater than 5 were included. The possible risk for publication bias was visualized using funnel plots. Figures referring to the analyses of the main study outcome (VAS results) are presented in the article. The plots do not show significant asymmetry. However, as the sample of the included articles was rather small, it is not possible to clearly assess the possibility of publication bias from the plots [42].

### 2.5. Outcome Measure

Assessment of the primary outcome included the visual analogue scale (VAS). Reported data for VAS were chosen post hoc as the primary outcome as the VAS scale is widely used in the literature and was the most commonly employed method in the articles included in the present review study. The secondary outcomes included the Tampa Scale for kinesiophobia (TSK), present pain intensity (PPI), Oswestry dysfunction index (ODI), and the neck disability index (NDI).

VAS measures pain intensity and patients rate pain intensity from 0–100 mm. The low score, i.e., (VAS: 0 mm), indicates no pain, and the high score (VAS: 100 mm) indicates severe pain. VAS’s minimal clinical important change (MCIC) shows a significant pain change if it varies by 25 mm. The VAS score was changed to 0–100 mm if VAS was measured at a different scale [43]. VAS and TSK have good validity and reliability in CLBP patients.

TSK is a subjective assessment of fear of re-injury and movement due to physical activity, measured using a 17-question inventory with answer possibilities ranging from 0–68. An high TSK score reflecting an high kinesiophobia [44].

The Oswestry Disability Index (ODI) and the Neck Disability Index (NDI) are questionnaires used to measure the permanent disability functions of patients for back and neck [45]. ODI consist of a 10-items for a total score from 0 to 100. No disability is represented by a score of 0–20, and a high level of disability is represented by a score of 80–100 [45]. NDI is measured using ten questions, where each one of the questions is answered on a 0–5 scale. The overall NDI questionnaire score range from a minimum of 0 to a maximum of 50. A low score represents a low neck disability while an a high score represents a high neck disability [46].

### 2.6. Data Synthesis and Statistical Assessment

Review Manager version 5.4.1. (The Cochrane Collaboration, Copenhagen, Denmark) was used to determine the pooled effect of primary and secondary outcomes obtained from eligible articles and used forest plots to present the outcomes [47]. The mean differences (MD) derived from the mean, standard deviation, and sample size were assessed. This analysis determined the overall mean difference, *p*-value at the level of significance, and heterogeneity. Mean values and 95% confidence intervals were reported. If the *p*-value was <0.05, the outcome was statistically significant. An I^2^ test assessed the heterogeneity level. The degree of heterogeneity was interpreted as: <25% low; 25% to 75% moderate; and I^2^ > 75% high. Random-effect models were used in the statistical analyses (due to the high heterogeneity in most of the analyses). Post hoc subgroup analyses were also conducted.

### 2.7. Risk of Bias (ROB) Assessment of Eligible Studies

ROB of eligible studies was measured using the Cochrane risk of bias assessment tool [48]. This tool evaluates bias for selection bias, reporting bias, performance bias, detection bias, attribution bias, attritions bias, and other bias of eligible articles. Each of the biases was classified as high-risk bias, low risk bias, and unclear.

## 3. Results

### 3.1. Literature Search and Characteristic of Study

**Figure 1:**
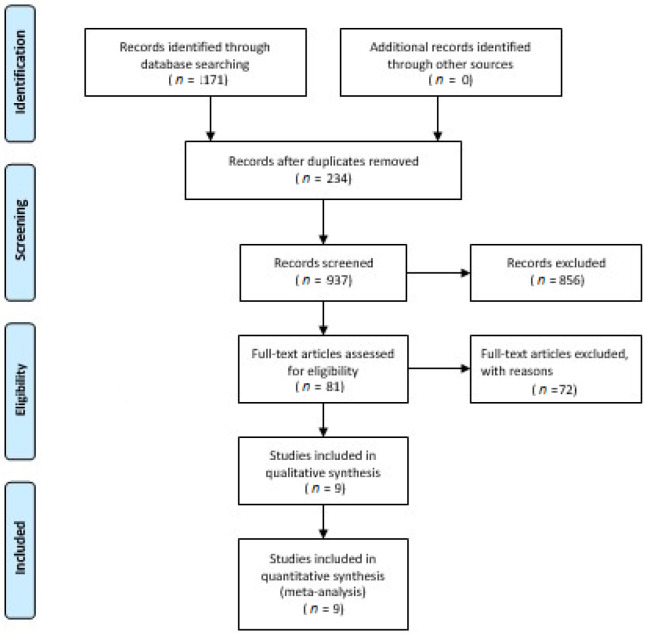
PRISMA flow diagram describing the article selection process.

Electronic literature searches using Boolean and keywords search identified 1171 unique records. Removal of 234 duplicate articles was performed using Endnote version 20 [49]. Eighty-one relevant articles were eligible for full-text inspection after screening 937 articles. After eliminating those articles according to the established criteria, nine articles reporting RCTs were included in the systematic review and quantitative meta-analysis. Figure 1 describes the flowchart of the inclusion method, following the PRISMA guidelines.

The nine studies that were deemed as eligible included a total of 524 chronic pain patients. Six of these studies investigated chronic low back pain [50,51,52,53] and three studies on chronic neck pain [54,55,56]. The characteristics of the studies included in the quantitative meta-analysis are summarized in Table 1.

### 3.2. Measure of Primary Outcomes

#### 3.2.1. Measure of VAS before and after Therapy

**Figure 2:**
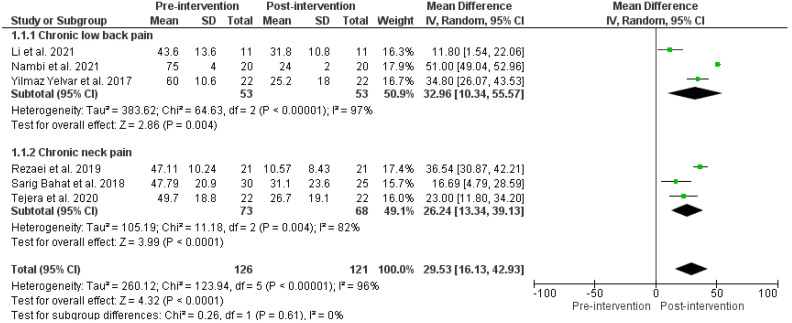
Effects before and after VR therapy on VAS score. Green squares represent mean difference for each study, while black rhombus represents the aggregated average of the mean differences.

Six studies compared VAS, before and after VR therapy. Lower VAS represented low levels of experienced pain. Three RCTs demonstrated significantly reduced VAS for chronic low back pain intensity following the VR-based therapy (MD VAS: 32.96 (10.34, 55.57), *p*-value < 0.05, I^2^ = 97%) [51,52,58]. Similarly, three RCTs demonstrated significantly reduced VAS for chronic neck pain intensity after VR therapy (MD: 26.24 (13.34, 39.13), *p*-value < 0.05, I^2^: 82%) [54,55,56]. The pooled VAS effect, before and after VR therapy, showed a significant reduction in pain intensity (MD: 29.53 (16.13–42.93), *p*-value < 0.05, I^2^ = 96), as shown in Figure 2. Heterogeneity was high among the overall effects of CLBP and chronic neck pain. Post hoc subgroup analyses showed low level of heterogeneity, suggesting that the clustering of different types of pain (neck and back pain) measures in the main analyses may be the cause of high heterogeneity in these analyses.

**Figure 3:**
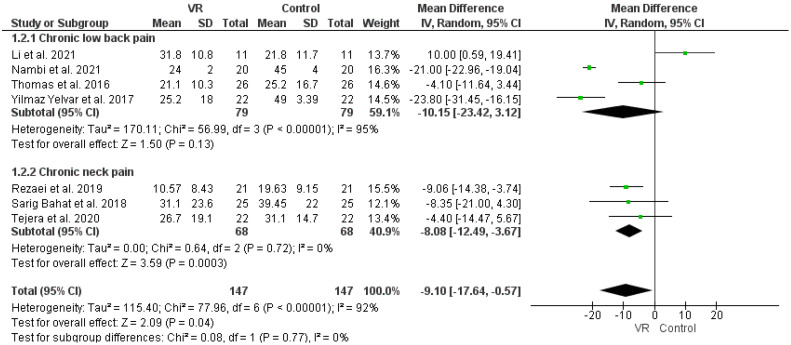
Effects of VR therapy and control on VAS score. Green squares represent mean difference for each study, while black rhombus represents the aggregated average of the mean differences.

The pooled analysis of four RCTs demonstrated insignificant improvements in the VAS of chronic low back pain with VR intervention compared to the control group (MD: −10.15 (−23.42, 3.12), *p*-value: 0.13, I^2^: 95%) [50,51,52,58]. However, the pooled VAS overall effects of three RCTs demonstrated significant improvements in chronic neck pain with VR therapy than in the control (MD: −8.80 (−12.49, −3.69), *p*-value < 0.05, I^2^: 0%) [54,55,56]. Furthermore, the overall effect in seven studies on pain demonstrated significant improvements in VAS effect with VR intervention (MD: −9.10 (−17.64, −0.57), *p*-value: 0.04, I^2^: 92%), as in Figure 3.

**Figure 4:**
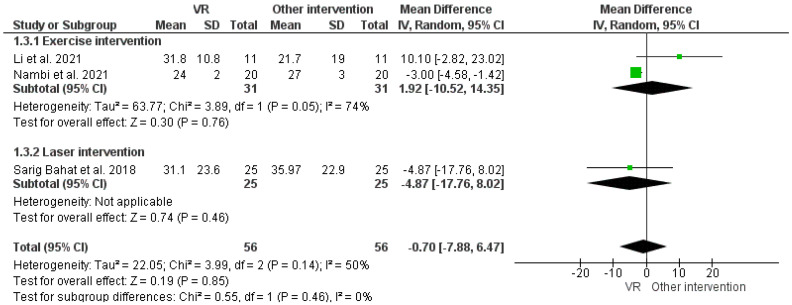
Effect of VR and other interventions on VAS improvement. Green squares represent mean difference for each study, while black rhombus represents the aggregated average of the mean differences.

**Figure 5:**
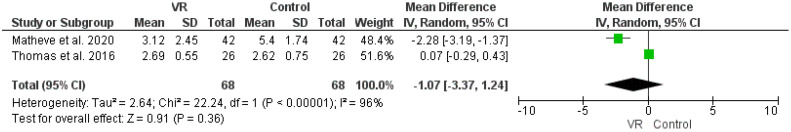
Effect of VR and control on PPI. Green squares represent mean difference for each study, while black rhombus represents the aggregated average of the mean differences.

The overall analysis of the VAS score for VR therapy with other interventions, i.e., exercise and laser therapy, demonstrated insignificant improvements in chronic pain, as shown in Figure 4. Similarly, the overall analysis of the PPI score with VR intervention over control therapy demonstrated an insignificant reduction in pain intensity, as shown in Figure 5.

#### 3.2.2. Measure of Secondary Outcome

##### Analysis of TSK

**Figure 6:**
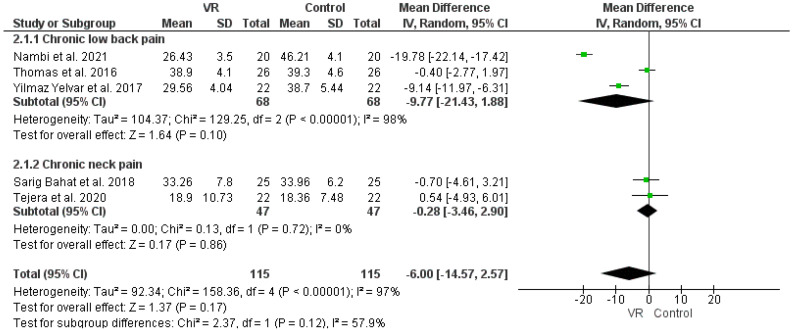
Effect of VR and control intervention on TSK outcome. Green squares represent mean difference for each study, while black rhombus represents the aggregated average of the mean differences.

The analysis of the TSK effect, as in Figure 6, was found to be neither clinically significant, nor statistically significant favorable for chronic low back pain (MD: −9.78 (−21.43, 1.88), *p*-value: 0.1), neck pain (MD: −0.28 (−3.46, 2.9), *p*-value: 0.86), or all cause of chronic pain (MD: −6.0 (−14.57, 2.57), *p*-value: 0.17).

##### Analysis of ODI

**Figure 7:**
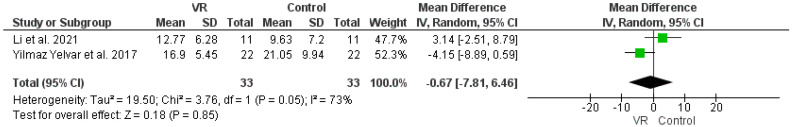
Effect of VR and control intervention on ODI outcome. Green squares represent mean difference for each study, while black rhombus represents the aggregated average of the mean differences.

The analysis of two RCTs demonstrated statistically insignificant favorable results of VR therapy in reducing ODI over care as usual (MD: −0.67 (−7.81, 6.46), *p*-value: 0.85 I^2^: 73%), as shown in Figure 7 [51,52].

##### Analysis of NDI

**Figure 8:**
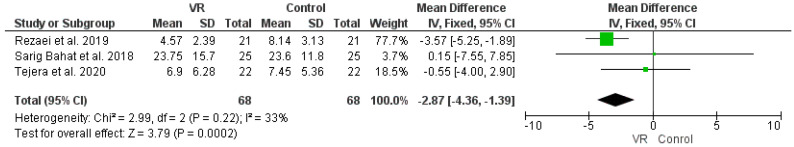
Effect of VR and control intervention on measure of ODI outcome. Green squares represent mean difference for each study, while black rhombus represents the aggregated average of the mean differences.

Analysis of three RCTs demonstrated a statistically significant favorable NDI effect due to VR therapy compared to care as usual (MD: −2.87 (−4.0, −1.39), *p*-value < 0.05 I^2^: 33%) as in Figure 8 [54,55,56].

### 3.3. ROB of Eligible Studies

**Figure 9:**
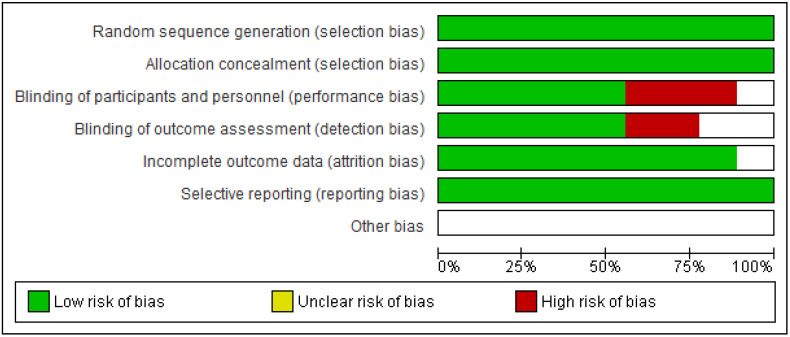
ROB graph.

**Figure 10:**
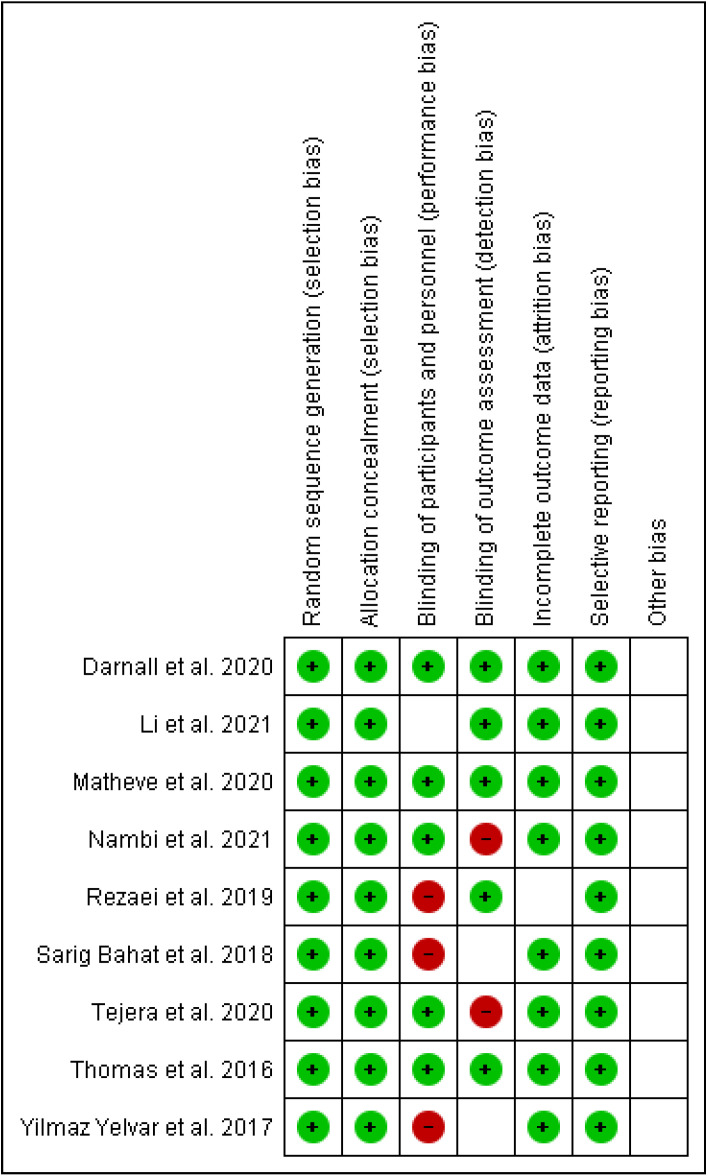
Summary of ROB for each included article.

**Figure 11:**
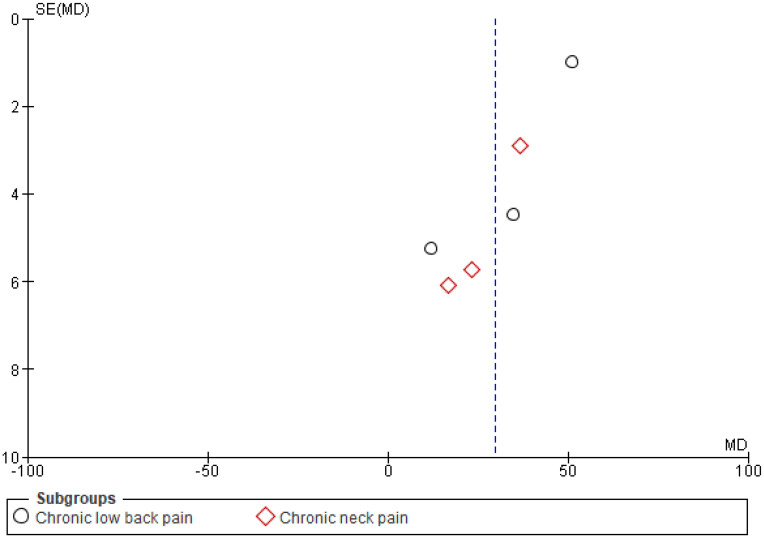
Funnel plot pre- and post-VR pain management intervention.

**Figure 12:**
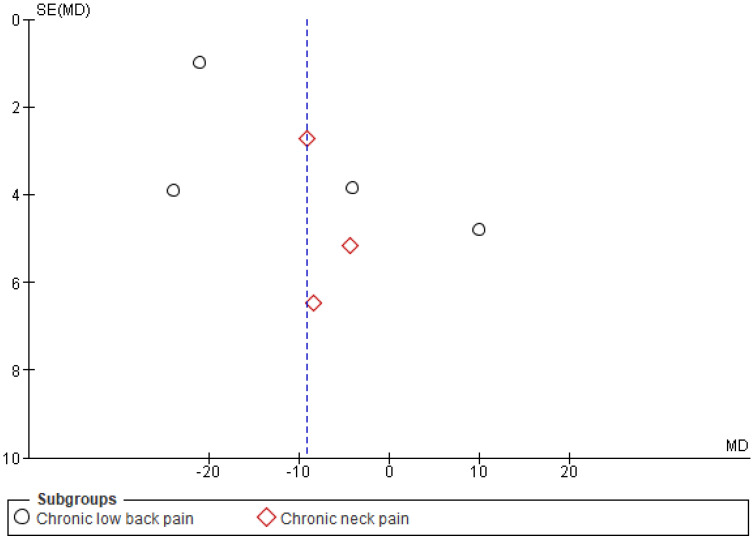
Funnel plot VR pain management intervention group vs. control group.

**Figure 13:**
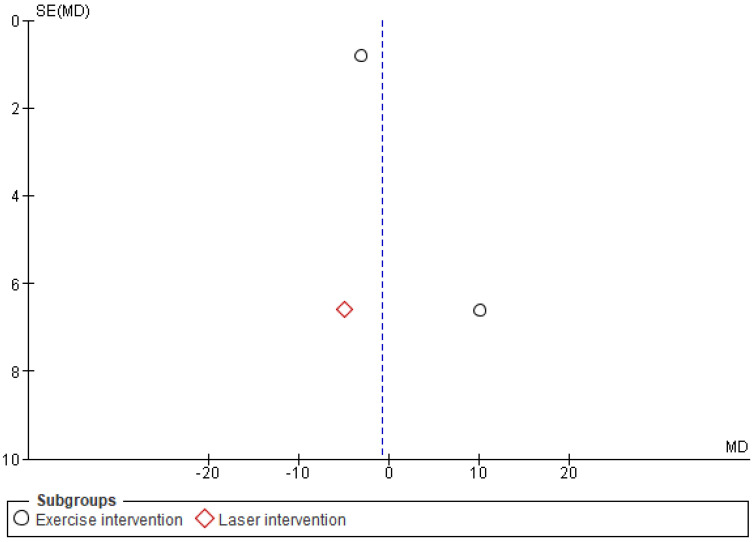
Funnel plot VR pain management intervention group vs. other pain management therapies group.

The Cochrane risk of bias assessment tool was used to assess the risk of bias of each included article. Figure 9 and Figure 10 present a graph and summary of the ROB assessment, respectively. Selection bias, attribution bias, and reporting bias were low in each study. Moreover, unclear risk of bias was observed for other biases in each included study. Three studies presented a high risk of bias [52,54,55]. Furthermore, two studies had a detection bias [56,58]. Figure 11, Figure 12 and Figure 13 show funnel plots computed using the VAS scores.

## 4. Discussion

The present systematic literature review and quantitative meta-analysis aimed to assess VR therapy effectiveness in the management of chronic pain. This was achieved firstly by individuating studies employing robust experimental methods (RCTs), evaluating the pre-therapy and post-therapy effects of VR-based treatments based on the scores of VAS. Finally, results from VR-based therapies were compared with results from other types (non-VR) of pain-management treatments, attempting to individuate a possible advantage or disadvantage of VR-based therapy compared to other types of used therapies for pain management.

The published scientific literature mentions the use of VR in the context of alleviation in neck pain [59], chronic lower-back pain (LBP) [50], sprained ankles, frozen shoulder, phantom limb syndrome, akinesia, phobias, ligament injuries, and treatment of physical dysfunction and anxiety [60,61]. The present systematic review and meta-analysis included nine studies. Of these studies, six studies measured pain intensity using VAS, before and after VR therapy [51,52,54,55,56,58]. Pain intensity was measured using either the VAS scale or PPI [43]. Seven studies measured VAS scale pain intensity. The pooled analysis of the VR effect on VAS demonstrated that VR-based therapies produced a significant improvement in pain symptoms. The outcomes of VR therapy over control demonstrated a significant reduction in pain perception in patients suffering from chronic neck pain [54,55,56]. However, the effect for CLBP was not statistically significant, despite following the same quantitative trend [50,51,52,58]. The study of Li et al. [51] reported that patients using VR therapy had worse quantitative outcomes compared to both motor control exercises and a control group; however, the data from the three groups did not differ statistically. The authors pointed to the relatively short duration of the pain-management intervention (reported duration of a total of two weeks with five days per week) as one of the possible causes for the lack of success of the interventions to decrease self-reported pain compared to the control.

A recent meta-analysis [62] showed the possible effect of VR-therapy for rehabilitation purposes. Such a finding is in contrast with the results reported in the present study. The present review differently from Bordeleau et al. [62] and includes newer studies and only includes RCTs. Furthermore, differently from previously published studies, the present review analyzes articles that specifically report cases of pain diagnoses, defined as chronic in the articles, or specifically referring to pain conditions with a duration of over 3 months.

According with the presented results, the effect of VR therapy on the reported VAS was not superior to other types of interventions in any of the analyzed metrics of pain severity. Similarly, no significant effects were observed for PPI between the VR and control groups.

### 4.1. Future Implication

Further research is required to provide a more definitive conclusion about VR effects in different chronic pain patients. Long-term therapeutic effects and follow-up analyses might allow to estimate the effect of VR-based therapies for prolonged pain relief. Future research may provide personalized therapeutic VR sessions to help patients with different needs to achieve therapeutic effects. More investigations should analyze pain-related outcomes as adverse events, returning to work from sickness leave, visits to healthcare professionals, and psychological health and well-being. Furthermore, future studies should investigate optimal VR exposure time for therapeutic uses.

Crucially, future scientific efforts should be focused on understand the mechanisms that allow VR-based therapies to be useful in chronic pain management, possibly employing physiological and neurophysiological indexes to identify biological mediating cause–effect relationships of VR-mediated therapeutic outcomes. In this context, the attention diversion hypothesis that has been proposed in the scientific literature [63] should be experimentally tested further. However, such a theory may find support from the ability that immersive VR has in subtracting attention from stimuli from the external world, which has been proven in neurophysiological experiments [64,65].

Gathering more evidence and developing guidelines and best practices for the use of VR in therapeutic settings has important practical implications. Small private clinics, hospitals, and other healthcare centers can easily be equipped with the technology to provide VR therapy to treat chronic pain patients [63]. Suitable VR systems are readily available due to recent technological advancements, and they are cost-effective, and one-time setup costs and training allow to provide repeated therapy.

### 4.2. Limitations

A major limitation of the present investigation is the small number of included studies. Only six studies assessed lower-back pain [50,51,52,53,57,58] and three studies measured VR effects on neck pain [54,55,56].

This study has small, pooled data due to a limited number of eligible studies that cause a lower power of overall effects. Additionally, substantial heterogeneity was present in most of the outcomes. Additionally, this review lacks measurements and comparisons of potentially important health-related outcomes of the different pain therapies, for example, stress, depression, and anxiety. However, none of the studies included in the review quantitatively measured and reported these outcomes and was out of the scope of the review.

In most of the included articles, VR-mediated intervention for pain management was used to assist with conventional non-pharmacologic treatments. Therefore, it is impossible to establish to what degree the therapeutic effects reported in the studies are imputable to the VR component of the therapy, or are, instead, attributable to the conventional component of the therapy (or to an interaction between the two components).

A single author has been working on all stages of the present study. This is certainly a limitation when considering the quality of data extraction, and possible biases in study selection and data interpretation.

## 5. Conclusions

There is limited evidence that supports the use of VR therapy in the context of chronic pain, despite rapid advancements in VR use in clinical settings. The present meta-analysis shows that VR interventions may be useful for chronic pain management. Especially, it shows as useful for neck pain and improved neck disability compared to controls. However, the analyses do not suggest the effectiveness of VR therapy over other types of treatments for pain management.

## Figures and Tables

**Table 1:** Characteristics of eligible studies.

Authors & Years	Study Design	Participants (I/C)	Age (I/C)	Pain Type	VR Environments	Treatment Conditions	Pain Assessment	Summary	NOS
Thomas et al. [50]	RCT	52 (26/26)	I: 23.9 ± 6.8 C: 26.7 ± 8.5	chronic low back pain	VR Dodgeball	IG: VR entertainment and distraction; CG: CAU	VAS, PPI, PRI, TKS	Decrease in VAS score & Pain intensity in VR group.	7
Li et al. [51]	RCT	34 (I: 11; EG: 12; C:11)	I: 21.91 ± 2.43 EG: 23.75 ± 4.09 CG: 25.36 ± 3.72	chronic low back pain	Kinect Xbox 360 system: Fruit Ninja game	IG: VR gaming + MT EG: Four-point kneeling exercise + MT CG:MT	VAS, ODI	VAS scores were reduced in each group after intervention.	6
Rezaei et al. [54]	RCT	42 (21/21)	I: 36.19 ± 9.80 C: 31.23 ± 9.49	chronic neck pain	Gaming: Cervi game	IG: VR gaming CG: CAU	VAS, NDI	VR improved neck pain and disability	7
Yilmaz Yelvar et al. [52]	RCT	44 (22/22)	I: 46.3 ± 3.4 C: 52.8 ± 11.5	chronic low-back pain	Video of walking down the Ireland forest	IG: virtual walking task CG: CAU	VAS, TSK, ODI	VR reduced VAS and TKS	8
Sarig Bahat et al. [55]	RCT	90 IG: 30 LG: 30 CG: 30	IG: 48 ± 5.49 LG: 47.6 ± 6.78 CG: 47.5 ± 6.9	chronic neck pain	VR: kinematic training	IG: kinematic training	VAS, NDI, TSK,	VR demonstrate improved pain relief	6
Matheve et al. [57]	RCT	84 (42/42)	I: 42.1 ± 11.5 C: 44.2 ± 11.9	chronic low back pain	VR gaming	IG: Exercise + VR CG: CAU	PPI	VR reduced PPI.	8
Darnall et al. [53]	RCT	74 (35/39)	18–74	chronic low back pain	VR multimedia with audio	IG: VR CG: audio effect	PPI	both groups demonstrated reduced pain effects	7
Tejera et al. [56]	RCT	44 (22/22)	I: 32.72 ± 11.63 C: 26.68 ± 9.21	chronic neck pain	VR Vox Play glass with HMD clamping system and smartphone + Full dive VR + VR Ocean Aquarium 3D	IG: VR Vox Play glass with HMD clamping system and smartphone CG: CAU	VAS, NDI, TSK,	VR did not demonstrate significant difference	8
Nambi et al. [58]	RCT	60 IG: 20 EG: 20 CG: 20	IG: 23.2 ± 1.5 EG: 22.8 ± 1.6 CG: 23.3 ± 1.5	chronic low back pain	VR shooting game	IG: VR + Shooting game EG: isokinetic exercise CG: conventional training	VAS, TSK	VAS and TSK reduced after VR therapy	6

TSK: Tampa scale for kinesiophobia, PPI: present pain intensity, PRI: pain rating index, RMD: Roland–Morris disability questionnaire, CAU: care as usual; MT: magnetic therapy; ODI: Oswestry dysfunction index; VAS: visual analog scale (0–100).

## Data Availability

Not applicable.
